# Pharmacokinetics and Biodistribution of *Rhopalurus junceus* Scorpion Venom in Tumor-Bearing Mice after Intravenous and Oral Administration

**DOI:** 10.29252/.23.4.287

**Published:** 2019-07

**Authors:** Alexis Díaz-García, Gilmara Pimentel González, Tais Basaco Bernabeu, Juan C. Rodríguez Aurrecochea, Hermis Rodríguez Sánchez, Iraida Sánchez Monzón, Maikel Hernández Gómez, Caridad Rodríguez Torres, Maria Regla Rodríguez Capote, Irania Guevara Orellanes

**Affiliations:** 1 Research Department, Laboratories of Biopharmaceuticals and Chemistries Productions (LABIOFAM), Havana, Cuba; 2 Radiopharmacy Laboratory, Oncology and Radiobiology National Institute, Havana, Cuba; 3 Research Department, Laboratory of Experimental pathology, Oncology and Radiobiology National Institute, Havana, Cuba; 4 Microbiology Department, Cell culture Laboratory, Tropical Medicine Institute “Pedro Kourí”, Havana, Cuba

**Keywords:** Intravenous, Mice, Oral administration, Radioactivity, Scorpion venom

## Abstract

**Introduction:**

*Rhopalurus junceus* scorpion venom has shown potential for anticancer treatment. However, there are no scientific evidence about venom pharmacokinetic (PK) and biodistribution (BD) in tumor-bearing mice.

**Methods:**

^131^I-labeled venom was administrated by intravenous (IV) and oral (PO) routes at the single dose of 12.5 mg/kg. Mice were sacrificed and blood samples, major organs, and tumor were taken at 10, 20, 40, 90, 180, 300, 480, and 1440 min.

**Results:**

For IV route, maximum peak concentration (C_max_), elimination half-lives, total body clearance (CL), distribution volume (V_d_), mean residence time (MRT), and area under curve (AUC) were 21.77 ± 2.45 %Dosis·h/mL, 12.65 ± 2.1 h, 4.59 ± 0.23 mL/h, 83.80 ± 12 mL, 12.49 ± 2.71 h, and 21.77 ± 2.45 %Dosis·h/mL, respectively. For PO route, they were 0.60 ± 0.07 %Dosis·h/mL, 9.33 ± 1.35 h, 36.94 ± 4.01 mL/h, 497.33 ± 30 mL, 12.40 ± 1.87 h, and 6.89 ± 1.18 %Dosis·h/mL, respectively. PK parameters (C_max_, CL, V_d_, and AUC) showed significant differences between IV and PO routes. Bioavailability was 31.6 ± 4% for PO dose. Kidney, stomach, liver, and lung for IV and stomach, kidney, spleen, and lung for PO routes showed the major uptakes for ^131^I-labeled venom. In tumor tissue, after the maximum uptake for both routes, there was a consistent behavior of radioactivity respect to the major organs during the first 480 min.

**Conclusion:**

The PK and BD of *R. junceus* venom in mice depend on the administration route. These data represent a starting point for future experiments with this scorpion venom in experimental models of cancer.

## INTRODUCTION

Scorpion venom is a mixture of different types of biologically active components. The biochemical characterization of scorpion venom has shown that it is composed of peptides and proteins of 60–70 amino acid residues, with cross-linked disulfide bridges acting mainly on ion channels present in cells from insects, crustaceous, and mammals[1,2]. These toxic peptides, acting alone or synergistically, are mainly responsible for the biological activity of scorpion venom either for defense from predators or capture the prey.

The therapeutic potential of scorpion venom comes from an increasing number of scientific records on antimicrobial, analgesic and antitumor effect of the whole venom and/or its purified compounds[3]. Experimental preclinical data has set the potential of scorpion venom in anticancer therapy as a result of the apoptotic and anti-proliferative properties of the venom along with the inhibitory effects on cancer progression. The anticancer property of scorpion venom has been studied in different types of cancers, including glioma, neuroblastoma, leukemia, and lymphoma, as well as breast, lung, and prostate cancers [3-5].

*Rhopalurus junceus* is a Cuban endemic scorpion extensively used in traditional medicine as pain relief, anti-inflammatory, and antitumor. Venom from this scorpion has recently been considered as an anticancer candidate due to its selective cytotoxic effect against epithelial cancer cell lines[6]. Additional studies from our group have reported that *R. junceus* scorpion venom is able to induce apoptotic cell death against cervical[6] and breast cancer[7] cell lines in *in vitro* experiments. In spite of these results, there is no scientific evidence on the behavior of *R. junceus* venom in tumor-bearing animals, their tissue distribution, and time-kinetic relationship. Therefore, the aim of the present study was to obtain evidence regarding the pharmacokinetic (PK) and biodistribution (BD) of *R. junceus* venom in tumor-bearing mice.

## MATERIALS AND METHODS

### Scorpion venom source

Venom was obtained from scorpions kept alive in the laboratories belonging to the Entrepreneurial Group of Biopharmaceuticals and Chemistries Productions (LABIOFAM), located in Havana, Cuba. Venom was extracted by electrical stimulation, dissolved in distilled water and centrifuged at 369 ×g for 15 min. The protein concentration in supernatant was calculated by Lowry’s method[8].

### Materials and reagents

Dulbecco’s modified Eagle’s medium (DMEM), L-glutamine, gentamicin, phosphate buffer, trichlroacetic acid, chloramine-T, and sodium metabisulfite were purchased from Sigma (St. Louis, MO, USA). Fetal bovine serum (FBS) was procured from Hyclone Laboratories Inc., USA. ^131^I-Na was obtained from Center of Isotopes (Havana, Cuba).

### Cells and animals

The mouse mammary adenocarcinoma cell line, F3II, was kindly provided by The Center of Molecular Immunology (Cuba). The cells were cultured and maintained in DMEM, supplemented with 10% heat-inactivated FBS, 2 mM of L-glutamine, and 80 μg/mL of gentamicin. Eight- to ten-week-old male BALB/c mice with the average weight of 20 ± 2 g were obtained from the National Center for Laboratory Animal Breeding (CENPALAB, Havana, Cuba). Mice were housed in controlled temperature and humidity. Food and water were administered *ad libitum*. The experimental procedure using animals was approved by the Institutional Committee for the Care and Use of Laboratory Animals (Protocol 2013/3) and performed in accordance with the EU Directive 2010/63/EU for animal experiments. For PK and BD studies, the mice were implanted with 2 × 10^5^/100 µl F3II m urine breast cancer cells by subcutaneous injection in the right flank. When tumor size became ~150 mm^3^, the animals were injected with a single intravenous (IV) or an oral (PO) dose.

### Radioiodination of the venom

Labeling of scorpion venom was carried out by the chloramin-T method[9]. This method specifically iodinates tyrosine residues in proteins, forming a stable covalent protein-^131^I bond. Briefly, 11 mg of scorpion venom was mixed with 400 µl of 0.5 M phosphate buffer, pH 7.2. Additionally, 400 μL of ^131^I-Na (4 mCi) and 100 µl of chloramine-T (6 mg/mL) were added to the mix sequentially. Finally, 150 µl of sodium metabisulfite (12 mg/mL) was added to stop the reaction. The pH control was achieved all the time by using buffers solution.

### Stability and efficiency studies of ^131^I-labeled *R. junceus* venom

Stability of the ^131^I-labeled *R. junceus* venom was determined *in vitr*o in mice serum, and normal saline was used as solvent for ascending thin layer chromatography (TLC) technique. The ^131^I-labeled *R. junceus* venom was mixed with freshly collected mice serum (1:9) and incubated in 1.5 ml-Eppendorf tubes at room temperature during 24 h. The samples were taken away at regular intervals up to 24 h, and then TLC was carried out. The TLC strips were counted for radioactivity in gamma ray spectrometer, and percentage labeling efficiency was calculated. Whatman paper descending chromatography was employed to monitor the labeling efficiency.

### Determination of PK parameters of ^131^I-labeled *R. junceus* venom

PK studies analyze two routes of administration, IV and PO. Forty mice were selected for each route of administration in tumor-bearing mice. The animals were separated into eight groups of five animals each and received a single-dose (12.5 mg/kg) of ^131^I-labeled-*R. junceus* venom with 3.67 MBq (400 µCi/mg) in a 100-µL volume. Mice were sacrificed at 10, 20, 40, 90, 180, 300, 480, and 1440 min following IV or PO administration. The time course of venom concentration in blood was followed by radioactivity. Whole blood samples were collected in 1.5-ml Eppendorf tubes for the measurement of radioactivity using a γ-scintillation counter CAPRAC-R (Capintec Inc, USA). The blood samples were centrifuged at 532 ×g at 4 °C for 10 min, and serum was obtained. The concentration of ^131^I-labeled-*R. junceus* venom in the serum was determined after trichlroacetic acid precipitation. In brief, serum samples (100 μL) from each animal were diluted separately in 400 μL of bovine serum albumin and then precipitated with 500 μL of 20% (v/v) trichlroacetic acid. Samples were incubated at 4 ºC for 30 min, and mixtures were centrifuged at 369 ×g at 4 °C for 15 min. Radioactivity in the pellet was measured in the γ-counter. Counts were corrected for background radiation, physical decay, and spillover of ^131^I counts. The results were presented as the percentage of injected dose (%ID)/ml blood vs. time. The data were fitted to non-compartment model for both routes of administration by using nonlinear regression in Gauss-Newton method in the computer software WinNonlin 5.0 (Pharsight Corporation, Mountain View, CA, USA). Non compartment equations were used to calculate various PK parameters, such as elimination half-life (T_½_), total body clearance (CL), distribution volume (V_d_), area under curve (AUC), maximum peak concentration (C_max_), time to reach the C_max_ (t_max_), and mean residence time (MRT). Bioavailability (F) of the PO dose of ^131^I-labeled-*R. junceus* venom respect to IV administration was calculated according to the formula F = AUC_PO_/AUC_IV_

### Biodistribution of ^131^I-labeled-venom

For BD studies, animal groups separated as described above were sacrificed and dissected at the time selected previously, after ^131^I-labeled-*R. junceus* venom administration by IV and PO routes. The whole blood, different organs such as heart, lung, liver, spleen, kidney, stomach, large intestine, small intestine, muscle, bone and tumor were removed, washed with normal saline and blotted. They were then weighed, and the corresponding radioactivity was measured using the γ-scintillation counter. The uptake of radioiodination in tissues and organs was expressed as %ID per gram of tissue (%ID/g).

### Production of specific anti-*R. junceus* IgG poly-clonal antibodies

Adult female New Zealand white rabbits (1.8–2 kg) were immunized by injecting subcutaneously one dose of 500 μg scorpion venom emulsified in 1 mL of complete Freund’s adjuvant. Three additional booster injections at 10-day intervals were performed with similar doses emulsified in 1 mL of incomplete Freund’s adjuvant. Ten days after the last injection, rabbits were bled by cardiac puncture. Serum was obtained, and anti-*R. junceus* IgG antibodies were purified by using affinity chromatography with a Sepharose 4B-CNBr-activated column as previously described[10].

### Simulated gastric juice (SGJ)

SGJ was based on studies carried out by Fu *et al*.[11] with slight modifications. Briefly, a hydrochloric acid solution was prepared, which included sodium chloride (2 mg/mL) and porcine pepsin (3.2 mg/mL) at pH 1.2. *R. junceus* scorpion venom was added at the final concentration of 1 mg/mL, and the final volume was 500 μL. In the experiment, a control solution without pepsin was included, and the incubation period was 20 min at 37ºC.

### Detection of *R. junceus* venom proteins in SGJ

After incubation period, SDS-PAGE analysis was performed under non-reducing conditions as previously described[12]. In brief, 50 μL of control and hydrochloric acid-treated samples was separated on a 16% SDS-PAGE gel. The separated bands were subsequently transferred to PVDF membranes, blocked with 5% skim milk and incubated with primary (anti-*R. junceus* IgG antibodies) and secondary anti-rabbit horseradish peroxidase-linked IgG antibody sequentially. Finally, bands recognized by antibodies were visualized by 3,3′-Diaminobenzidine staining.

### Statistical analysis

For statistics, we used the GraphPad Prism version 5.01 for Windows, (GraphPad Software, San Diego California, USA). All values were presented as mean ± standard deviation. Comparison of PK parameters between IV and PO routes was analyzed by the two-tailed unpaired or paired student’s *t*-test. For all the analyses, *p* < 0.05 was considered statistically as significant.

## RESULTS

**Fig. 1:**
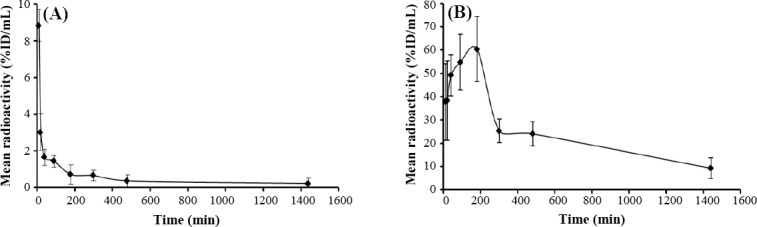
Serum concentration-time profile of ^131^I-labeled *R. junceus* scorpion venom after (A) intravenous and (B) oral administration of 12.5 mg/kg in F3II tumor-bearing mice. Data are reported as means ± SD (n = 5).

To determine the PK and BD properties *in vivo*, *R. junceus* scorpion venom was radiolabeled with iodine-131. The radioiodination method achieved more than 95.2 ± 0.3% of labeling. The stability in *in vitro* assay showed that ^131^I-labeled scorpion venom was stable in mice serum at least until 24 h. Because the labeling efficiencies exceeded 95%, no further purification was performed for animal studies. Measurements of radioactivity in whole blood and serum showed differences, with higher radioactivity measurements in serum respect to whole blood (data not shown). The mean radioactivity (% ID/ml) in serum versus time curve after IV administration in tumor-bearing mice is shown in Figure 1A, which shows a fast distribution phase to tissue from the beginning until 40 min and a second slow elimination phase. The PO administration showed a typical curve with an initial absorption phase lasting around 3 h, a rapid distribution phase taking two hours and a third slow elimination phase until the end of measurements (Fig. 1B).

The PK parameters from both routes of administration are shown in Table 1. For IV route, C_max_ (27.647 ± 6.345 %Dosis/mL) showed significant difference (*p* < 0.05) respect to PO route C_max_ (0.60 ± 0.07 %Dosis/mL), which was 46 times higher. Meanwhile, AUC from IV and PO routes showed significant differences (*p* < 0.05); the AUC_IV_/AUC_PO_ value was 3.1 times (Table 1). The PO elimination rate (36.94 ± 4.01 mL/h) showed significant differences (*p* < 0.05) respect to IV route (4.59 ± 0.23 mL/h). The apparent volume in PO route also displayed significant differences (*p* < 0.05) respect to IV route (Table 1). In both routes of administration, the MRT was relatively similar (Table 1). At the same time, the T½ in IV was 12.65 ± 2.1 h, but that of PO was 9.33 ± 1.35 h, which represent only 1.33 times higher. The PO bioavailability of scorpion venom was 31.6 ± 4%.

**Table 1:** Pharmacokinetic (PK) parameters of ^131^I-labeled-*R. junceus* venom after intravenous (IV) and oral (PO) administration at 12.5 mg/kg of scorpion venom in tumor-bearing mice.

PK parameters	IV	PO
AUC (%Dosis·h/mL)	21.77 ± 2.45^*^	6.89 ± 1.18
CL (mL/h)	4.59 ± 0.23^*^	36.94 ± 4.01
V_d_ (mL)	83.80 ± 12.00^*^	497.33 ± 30.00
T½ (h)	12.65 ± 2.10	9.33 ± 1.35
MRT (h)	12.49 ± 2.71	12.40 ± 1.87
Cmax (%Dosis/mL)	27.64 ± 6.34^*^	0.60 ± 0.07
T max (h)	0.00	3.00 ± 0.30

Significant differences

* *p* < 0.05 respect to PO route

**Fig. 2:**
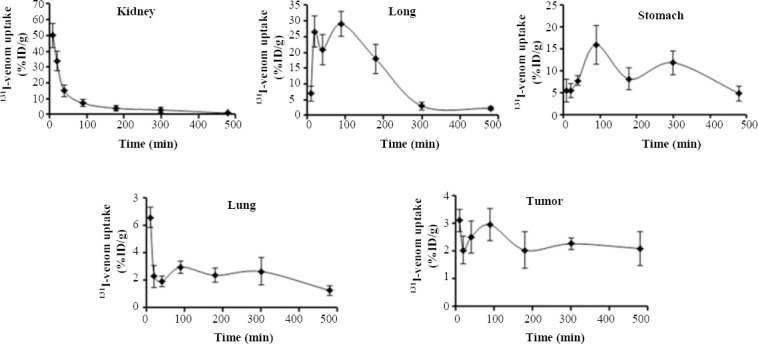
Uptake kinetic of ^131^I-labeled *R. junceus* venom in the major four organs with highest radioactivity and tumor tissue in F3II tumor-bearing mice after the first 480 min. Eight- to ten-week-old mice were injected intravenously with 12.5 mg/kg of ^131^labeled-venom. At designated time points, the mice were sacrificed by cervical dislocation and dissected. Radioactivity associated with the tumor and normal tissues was measured. Data were reported as means ± SD (n = 5). Bars illustrate standard deviations.

The radioactivity of ^131^I-labeled *R. junceus* venom in tissue distribution from IV and PO routes was determined at different time intervals. The BD of ^131^I-labeled *R. junceus* venom in the major organs after IV injection is shown in Table 2. During the first two hours, a relatively high uptake of ^131^I-labeled *R. junceus* venom was observed in those organs that are well vascularized and involved in biotransformation and excretion processes, such as stomach, kidney, liver, and lung (Table 2). From IV route, the radioactivity revealed that 10 min after the radioconjugate injection, the kidney contained the highest radioactivity with 49.94 ± 7.69 %ID/g. Besides, lung was the second organ with 29.01 ± 11.98 %ID/g, and stomach was the third with 15.96 ± 6.37 %ID/g, both at 1.5 h. Finally, liver showed 6.60 ± 0.76 %ID/g at 10 min after venom injection (Table 2). Tumor tissue demonstrated a maximum peak of 3.11 ± 0.40 %ID/g at 10 min after IV injection (Table 2). In all four organs, after radioactivity reached the maximum, the values decreased in a time-dependent manner (Fig. 2). Only in tumor tissue, after the maximum uptake, there was a consistent behavior in terms of radioactivity (~2 %ID/g), during the first 480 min (Fig. 2)

**Table 2:** Radioactivity in organs after intravenous injection of ^131^I-labeled *R. junceus* venom in F3II tumor-bearing mice

Organ	10 min	20 min	40 min	90 min	180 min	300 min	480 min	1440 min
Blood	5.09 ± 1.00	3.54 ± 0.63	3.29 ± 0.51	3.83 ± 0.12	2.34 ± 0.92	2.77 ± 0.44	1.77 ± 0.34	0.13 ± 0.04
Heart	2.71 ± 0.61	2.29 ± 0.84	2.07 ± 0.45	2.48 ± 0.68	1.46 ± 0.50	1.36 ± 0.29	0.29 ± 0.02	0.07 ± 0.00
Lung	6.93 ± 2.47	26.62 ± 11.06	14.84 ± 8.75	29.01 ± 11.98	17.95 ± 9.72	2.96 ± 0.08	2.22 ± 0.63	2.23 ± 1.17
Liver	6.60 ± 0.76	2.36 ± 0.80	1.91 ± 0.38	2.97 ± 0.46	2.37 ± 0.52	2.65 ± 1.63	1.26 ± 0.35	0.43 ± 0.08
Spleen	2.53 ± 0.10	2.03 ± 0.69	1.74 ± 0.38	2.89 ± 0.37	2.42 ± 0.78	1.46 ± 0.45	0.50 ± 0.09	0.63 ± 0.03
Kidney	49.94 ± 7.69	33.82 ± 9.42	15.04 ± 1.66	7.11 ± 2.20	3.84 ± 1.59	2.81 ± 0.68	0.81 ± 0.23	0.30 ± 0.05
Stomach	5.51 ± 2.57	5.47 ± 1.63	7.75 ± 1.08	15.96 ± 6.37	8.15 ± 2.51	11.85 ± 2.73	4.85 ± 1.73	0.81 ± 0.22
LI	3.28 ± 1.94	3.12 ± 0.89	2.26 ± 0.09	2.68 ± 0.51	4.43 ± 0.36	1.56 ± 0.23	0.86 ± 0.18	0.11 ± 0.02
SI	1.67 ± 0.86	1.84 ± 0.81	1.61 ± 0.33	2.49 ± 0.29	1.86 ± 0.58	1.81 ± 0.12	1.10 ± 0.33	0.08 ± 0.01
Bone	1.38 ± 0.24	1.39 ± 0.41	1.73 ± 0.48	2.20 ± 0.39	1.51 ± 0.49	1.47 ± 0.31	0.86 ± 0.37	0.10 ± 0.02
Muscle	1.20 ± 0.47	0.64 ± 0.14	0.73 ± 0.04	0.86 ± 0.05	1.01 ± 0.18	0.78 ± 0.15	0.34 ± 0.09	0.05 ± 0.03
Tumor	3.11 ± 0.40	2.03 ± 0.80	2.51 ± 0.58	2.96 ± 0.59	2.04 ± 0.67	2.27 ± 0.20	2.10 ± 0.61	0.18 ± 0.07

Values are presented as the percentage of injected dose per gram (% ID/g, mean ± SD, n = 5 at each time point)

Table 3 depicts the tissue distribution of ^131^I-labeled *R. junceus* venom after PO administration. Only stomach and kidney showed radioactivity higher than 1 %ID/g with 8.60 ± 2.26 and 1.68 ± 0.45 %ID/g at 20 min and 40 min, respectively. Besides, spleen and lung were the third and four major organs with the highest radioactivity (0.72 ± 0.09 and 0.64 ± 0.19 %ID/g, respectively) at 40 min later. Tumor tissue exhibited its highest uptake (0.27 ± 0.06 %ID/g) after 40 min of ^131^I-*R. junceus* venom administration (Table 3).

**Table 3:** Radioactivity in organs after oral administration of ^131^I-labeled *R. junceus* venom in F3II tumor-bearing mice

ORGAN	10 min	20 min	40 min	90 min	180 min	300 min	480 min	1440 min
Blood	0.61 ± 0.2	0.81 ± 0.05	0.86± 0.19	0.72 ± 0.12	0.55 ± 0.19	0.57 ± 0.03	0.40 ± 0.12	0.04 ± 0.02
Heart	0.30 ± 0.1	0.32 ± 0.07	0.27 ± 0.07	0.24 ± 0.1	0.19 ± 0.03	0.15 ± 0.01	0.18 ± 0.02	0.03 ± 0.03
Lung	0.34 ± 0.09	0.5 ± 0.16	0.64 ± 0.19	0.41 ± 0.12	0.39 ± 0.06	0.34 ± 0.067	0.35 ± 0.06	0.04 ± 0.01
Liver	0.33 ± 0.10	0.35 ± 0.07	0.33 ± 0.12	0.22 ± 0.10	0.19 ± 0.09	0.17 ± 0.09	0.16 ± 0.02	0.03 ± 0.00
Spleen	0.28 ± 0.07	0.44 ± 0.12	0.72 ± 0.09	0.46 ± 0.10	0.40 ± 0.17	0.41 ± 0.09	0.49 ± 0.13	0.06 ± 0.02
Kidney	0.28 ± 0.07	0.87 ± 0.20	1.68 ± 0.45	1.39 ± 0.36	1.81 ± 0.27	1.81 ± 0.42	1.95 ± 0.45	0.09 ± 0.02
Stomach	2.92 ± 0.91	8.60 ± 2.26	6.09 ± 1.78	2.23 ± 0.37	0.99 ± 0.26	0.49 ± 0.86	0.48 ± 0.16	0.08 ± 0.02
LI	0.26 ± 0.06	0.41 ± 0.13	0.44 ± 0.08	0.24 ± 0.04	0.28 ± 0.07	0.37 ± 0.04	0.42 ± 0.12	0.03 ± 0.01
SI	0.18 ± 0.00	0.34 ± 0.14	0.33 ± 0.08	0.27 ± 0.06	0.23 ± 0.05	0.27 ± 0.05	0.24 ± 0.02	0.03 ± 0.03
Bone	0.18 ± 0.04	0.19 ± 0.04	0.4 ± 0.09	0.29 ± 0.1	0.26 ± 0.06	0.19 ± 0.09	0.17 ± 0.00	0.06 ± 0.02
Muscle	0.1 ± 0.07	0.12 ± 0.04	0.14 ± 0.03	0.10 ± 0.05	0.09 ± 0.00	0.06 ± 0.01	0.10 ± 0.03	0.03 ± 0.00
Tumor	0.11 ± 0.04	0.16 ± 0.02	0.27 ± 0.06	0.22 ± 0.09	0.25 ± 0.05	0.23 ± 0.03	0.20 ± 0.05	0.02 ± 0.02

Values are presented as percentage of injected dose per gram (% ID/g, mean ± SD, n = 5 at each time-point)

**Fig. 3:**
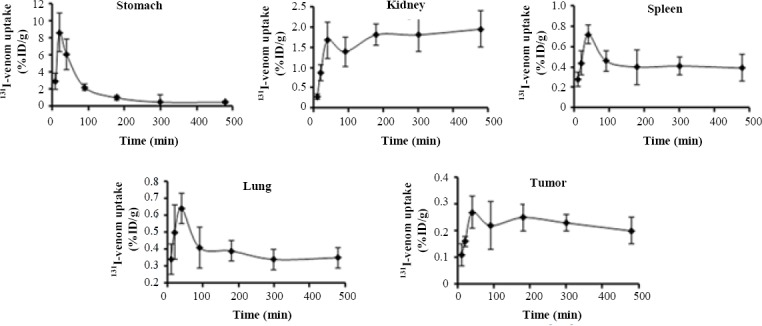
Uptake kinetic of ^131^I-labeled *R. junceus* venom in the major four organs with highest radioactivity and tumor tissue in F3II tumor-bearing mice after the first 8 h. Eight- to ten-week-old mice were administered 12.5 mg/kg, orally. At designated time points, the mice were sacrificed by cervical dislocation and dissected. Radioactivity associated with the tumor and normal tissues was measured. Data are reported as means ± SD (n = 5). Bars illustrate standard deviations.

**Fig. 4:**
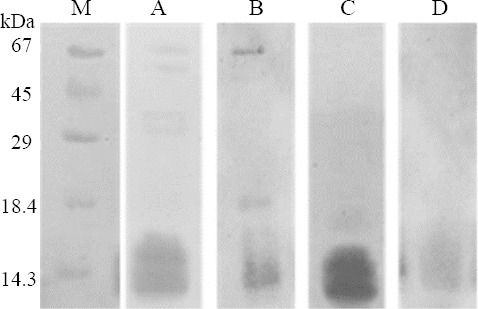
Non-reduced SDS-PAGE and Western blot of *R. junceus* scorpion venom. Lane A, SDS-PAGE of untreated scorpion venom; lane B, Western blot of untreated scorpion venom; lane C, SDS-PAGE of simulated gastric juice-treated scorpion venom; lane D, Western blot of SGJ-treated scorpion venom (lane D). M, molecular marker

By PO route, similar to IV route, three out of four major organs in terms of radioactivity reached the maximum value and then displayed a decreased along the time (Fig. 3). Only kidney tissue showed a consistent radioactivity uptake (Fig. 3). Similarly, after maximum uptake in tumor tissue, there was a consistent behavior in terms of radioactivity (~0.2 %ID/g; Fig. 3). In PO administration, the gastrointestinal tract represents an aggressive environment. In this sense, we studied the potential degradation process of scorpion venom in SGJ assay. After 20 min of incubation, control untreated and hydrochloric acid samples were analyzed by SDS-PAGE and Western blot using anti-*R. junceus* IgG antibodies. Figure 4 (lane A) shows the SDS-PAGE of *R. junceus* venom in untreated sample with protein bands at 67 kDa, between 45 kDa, and 29 kDa, as well as at 18.4 kDa and 14.3 kDa.

Western blot revealed that anti-*R. junceus* IgG antibodies was able to recognize protein bands in control untreated samples (Fig. 4, lane B). Meanwhile, scorpion venom in SGJ did not show any protein bands at 67 kDa, 45 kDa, 29 kDa, and 18.4 kDa, but at the 14.3 kDa band (Fig. 4, lane C). Western blot using specific anti-*R. junceus* IgG antibodies could only recognize low molecular proteins bands in SGJ-treated scorpion venom (Fig. 4, lane D).

## DISCUSSION

*R. junceus* scorpion venom has shown potential as an anticancer natural product due to cytotoxic selective effect against epithelial cancer cells *in vitro*. Tissue distribution and kinetic experiments of this venom could be a starting point to understand its behavior in the organism.. For this purpose, ^131^I-labeled *R. junceus* venom was administered by IV and PO routes in tumor-bearing mice.

The radioiodination has previously been used successfully for PK and BD studies of venoms[13]. In PK studies, scorpion venom radioiodination has been useful to describe the PK behavior after parenteral injection and to explain the ineffectiveness of some antisera, after envenomation, mainly due to delay in antiserum administration[14]. Meanwhile, in BD studies, the use of radioiodination has been able to identify the primary sites of venoms action[15]. In our experiments, in IV route, the initial phase is characterized by fast distribution preceded by short distribution half-life of *R. junceus* scorpion venom. This characteristic has been seen in different scorpion venoms[16,17]. This rapid process is due to scorpion toxins and is in accordance with the rapid appearance of sign and symptoms in the stung victims[18,19]. In PO administration, considering T_max_, the absorption of venom was slow, followed by a slow distribution phase compared to IV route. This trend of toxin distribution in PO rout may reduce the toxic effects or delay any potential pharmacological effect compared to other administration routes.

In the experiments, the elimination half-life shows to be slow (T½ > 8 h) and similar in both cases (IV and PO routes). This behavior is the same as some other scorpion venoms with elimination half-life between 6-24 h[20]. The V_d_, in both cases, were higher than the total blood volume of mice, showing that this scorpion venom can reach all tissue of the animals as was detected during BD experiments, in all selected organs. The highest uptake in each organ was different when comparing both administration routes. These results indicate that the PK profile and the main target organs depend on the administration route of the venom. This characteristic is similar to other compounds evaluated by the same administration routes in our study[21].

On other hand, differences were observed in the clearance kinetics of radioactivity in kidney, after PO administration. This observation represents a particular

finding in our experiment. In PO route, starting from maximum peak concentration until 480 min, kidney uptake was consistent. Some researchers have reported that kidney uptake is influenced by the interactions of radiopharmaceuticals with renal compartments such as the glomerulus and proximal tubules. The uptake of the radiolabeled compound into cells of the proximal tubules is subject to different mechanisms depending on the total charge, distribution of charge, and structural size[22]. Peptides and small molecules often interact with cells of the proximal tubules, resulting in effective reabsorption and renal trapping of the radiopharmaceutical[23]. Eder *et al*.[24] have shown that lysine positively charged peptides augment their kidney binding. One explanation for this observation would be the exposure of negatively charged sites located on the membranes of renal tubular cells which facilitate the accumulation of radiopharmaceutical peptides in the kidneys. Scorpion venom is a complex mixture of positively charged peptides acting mainly on ion channels and/or membrane receptors[1,25,26]. The PO administration of ^131^I-labeled venom can generate a high proportion of peptide fragments bounded to ^131^I. These ^131^I-labeled fragments, along with the remaining ones, can enhance the renal uptake of toxins and keep them for a longer period in the proximal tubules in kidney. In both administration routes, kidney, lung, and stomach showed the highest radioactivity.

By IV route, after reaching maximum peak concentration of ^131^I-labeled scorpion venom in tumor tissue, the uptake was similar during the experiment (~2 %ID/g). This characteristic was the same as PO where radioactivity was low but consistent in tumor (~0.2 %ID/g). This low uptake agrees with the PO bioavailability of scorpion venom of around 30% compared to IV route, indicating a massive loss of venom components. After PO administration, there was a lower percentage of ^131^I-venom proteins present in blood than that observed by endovenous route. This means that a large proportion of ^131^I-labelled venom has been degraded before and during absorption process in gut when administered PO. This degradation occurred because of harsh environment of GI tract[1,25], inducing dehalogenation and releasing ^131^I- and

^131^I-containing fragments[20]. Gastric mucosa has the capacity to actively accumulate free iodine. The constitutive expression of gastric sodium/iodine symporter in membranes of gastric cells mediates the active uptake of free iodine in gastric environment and from the bloodstream into the gastric lumen (gastric juice). The iodine concentrated in the gastric juice is excreted by glomerular filtration through the kidney[27].

Based on the above facts, after PO administration, the counts obtained from all tissues analyzed in the study could, with high probability, match to ^131^I-venom components/fragments. Besides, a previous report has evaluated the behavior of large proteins (150 kDa) during PO administration and identified that ^131^I-labelled large proteins are degraded, and ^131^I-protein fragments less than 12 kDa are able to traverse the gastrointestinal tract and appear in blood[28]. Additionally, Wang *et al*.[29] have evaluated the stability of larger, small and cyclic peptides in gastric and intestinal fluids *in vitro*. The authors concluded that peptide stability varies widely depending on the amino acid sequence and structures. Cyclic peptides become the most stable peptides because disulfide bond linkages offer high rigidity and low flexibility, which provide resistance against enzymatic cleavage of susceptible peptide bonds. However, larger peptides are more vulnerable[29].

Scorpion venom consists of various proteins and peptides. Proteins include enzymes such as phospholipase, hyaluronidase, L-amino acid oxidase, metalloproteinase, serine protease, and muco-proteins[25]. The highest proportion of scorpion venom components belongs to peptides less than 8 kDa with three to four disulfide bridges that target sodium, calcium, and potassium ion channels. There are also some anionic and antimicrobial peptides without disulfide bonds with a high frequency in scorpion venom[26,30]. The presence of disulfide-bridged in the peptides makes them more stable and less accessible to enzymatic cleavage[29,31]. Then, after PO administration, some ^131^I-venom components would pass through the gastrointestinal tract and reach the blood circulation in intact form due to the low molecular weight and possibly a strong secondary structure characterized by 3-4 disulfide bonds. Even after degradation process, some ^131^I-labeled fragments could keep their biological effect and reach target organs/tumor. Vallespí *et al*.[32] have assessed the PK and BD behaviors of CIGB-552, as an antitumor peptide, on tumor-bearing BALB/c mice. Experiments have shown that the degradation pattern of CIGB-552, based on serine-proteases activities in blood generates peptide metabolites that still keep the cytotoxic effect against tumor cells. The presence of consistent radioactive counts in tumor after PO administration suggests that a low proportion of undegraded ^131^I-venom components or ^131^I-labelled fragments might be able to reach tumor tissue, as was evidenced in BD. This observation suggests that tumor-targeting properties of scorpion venom, by PO, were not totally affected respect to IV route. Additionally, Krifi *et al*.[20] compared radioiodination and ELISA methods in PK studies using a low-molecular-weight fraction from the scorpion venom *Androctonus australis*. Authors concluded that antibody recognition-based methods, like ELISA, could distinguish degraded and native toxins as an advantage respect to radioactivity methods.

Based on these previous statements, we carried out SGJ experiments to follow fate of scorpion venom in the gastric environment. Using Western blot analysis, we showed that IgG-anti *R. junceus* venom still recognize some components from scorpion venom, in the low-molecular-weight zone, suggesting the presence of a low proportion of native peptides probably disulfide-bridged containing peptides among others. Moreover, previous studies by our group and others, described the presence of hyaluronidases around 45 kDa in *R. junceus* scorpion venom determined by SDS-PAGE[12,33]. These enzymes are partially responsible for toxicity related to lactate dehydrogenase and creatine kinase release, when scorpion venom is administrated by parenteral route in mice[12]. In SGJ, the 45-kDa band was lost due to the presence of pepsin and acidic environment, which indicates a reduction in toxic components of scorpion venom by PO. On other hand, in major organs like kidney, lung, and stomach, radioactivity reached the maximum and decreased rapidly, after IV and PO administration. This behavior suggests that some scorpion venom components have higher affinity to tumor tissue, which is in agreement with our previous results from *in vitro* experiments where scorpion venom showed to be selective for epithelial cancer cells respect to normal counterpart[6].

To our knowledge, the venoms from other scorpion species have never been evaluated for toxicological or pharmacological properties by PO. The base of our particular case comes from the traditional medicine in Cuba, where this endemic scorpion, *R. junceus*, has popularly been used by PO. By PO administration or IV injection, the *R. junceus* scorpion venom can reach all tissues in the body. The scorpion venom has a long-lasting presence in tumor tissue compared to main organs where scorpion venom presence is transient. The PO absolute bioavailability of scorpion venom is low, which suggests high degradation of venom components but still keeping the tumor-targeting properties. The knowledge of differences between PO and IV routes should be considered for future experiments in toxicological and pharmacological experimental models. The PK and tissue distribution results represent a starting point for future experiments with this scorpion venom as a potential anticancer natural extract.
